# Translation and manipulation of silicon nanomembranes using holographic optical tweezers

**DOI:** 10.1186/1556-276X-6-507

**Published:** 2011-08-25

**Authors:** Stefan M Oehrlein, Jose R Sanchez-Perez, RB Jacobson, Frank S Flack, Ryan J Kershner, Max G Lagally

**Affiliations:** 1 University of Wisconsin, Madison, WI 53706, USA

**Keywords:** optical trapping, silicon nanomembrane, nanofabrication, directed assembly

## Abstract

We demonstrate the use of holographic optical tweezers for trapping and manipulating silicon nanomembranes. These macroscopic free-standing sheets of single-crystalline silicon are attractive for use in next-generation flexible electronics. We achieve three-dimensional control by attaching a functionalized silica bead to the silicon surface, enabling non-contact trapping and manipulation of planar structures with high aspect ratios (high lateral size to thickness). Using as few as one trap and trapping powers as low as several hundred milliwatts, silicon nanomembranes can be rotated and translated in a solution over large distances.

## Introduction

Silicon nanomembranes are flexible, single-crystalline sheets with thicknesses ranging from less than ten up to several hundred nanometers [1,2]. These materials are extremely attractive for use in fast-flexible-electronic, optoelectronic, and nanophotonic applications. This broad potential derives from the unique properties imparted by the membranes' thinness relative to silicon wafers, including robustness, flexibility, and bondability. The structures can also be strain engineered to enhance individual electronic and mechanical properties or to produce unique tubular and helical nanostructures [2-6]. Successful integration of these structures into next-generation devices will require new paradigms for their assembly. The most promising methods for transferring and manipulating silicon nanomembranes to date include wet transfer (whereby nanomembranes are moved from the original substrate in a solution via adhesive attachment to a new host), dry transfer, and stamp printing processes [7-9] As nanomembranes are made thinner and thus become more difficult to handle, mechanical means of manipulation are limited in their precision with regards to controllably placing individual membranes.

Holographic optical trapping [10-13] offers a promising new approach for manipulating silicon nanomembranes with a high degree of accuracy and precision that may circumvent some of the above issues. Optical tweezers use a single, tightly focused beam of light to manipulate micro- and nanoscale objects in three dimensions. The technique enables precise positional control in a non-contact and non-invasive fashion without damage to the trapped object [14]. Holographic optical trapping uses an array of traps to extend these same capabilities to multiple points in space. Each trap can be independently and dynamically controlled in real time. Although holographic optical tweezers have been used to manipulate microspheres, nanowires, and arbitrarily shaped biological molecules [15-18] application to two-dimensional (planar) geometries has been limited to objects with low aspect ratios and with dimensions less than approximately 5-10 × 5-10 μm [19,20] The nanomembranes used for this work have thicknesses of 220 nm and in-plane dimensions of 50 × 50 μm, giving edge length-to-thickness aspect ratios of over 200. We believe this is the first time high-aspect-ratio planar materials have been successfully manipulated using non-contact techniques.

Most recent work on trapping and manipulating semiconductor materials has focused on one-dimensional nanowires [21,22], where the large index of refraction mismatches with the fluid medium complicates direct manipulation with the beam. Nanowires also tend to align along the axis of the laser and can be completely ejected from the trap, because of the overwhelming scattering force. This problem was recently circumvented for high-index vanadium oxide nanowires [23] using silica beads as a handle, the optical trapping of which has been well described in the literature [24]. The attachment of beads is commonly used when working with biological materials, where the arbitrary shape of the molecule or damage from laser heating can preclude trapping [25,26]. Wider application to directed assembly is limited, however, because removal of the handle has remained an issue: the manipulated object needed to be laser cut or damaged to achieve detachment or in other cases the covalently bonded bead could not be detached at all.

We present here a significant step forward, extending this initial work to the silicon nanomembranes described above. By attaching a single bead to the edge of a membrane, we achieve full three-dimensional control of large-area planar objects using optical tweezers. We furthermore succeed in reversible detachment of the handle bead without damage to the membrane, providing a substantial advantage over previous work. These capabilities will enable new, flexible routes for assembly of a variety of two-dimensional thin sheets of direct relevance to semiconductor, biotechnology, and sensor technologies.

## Results and discussion

**Figure 1:**
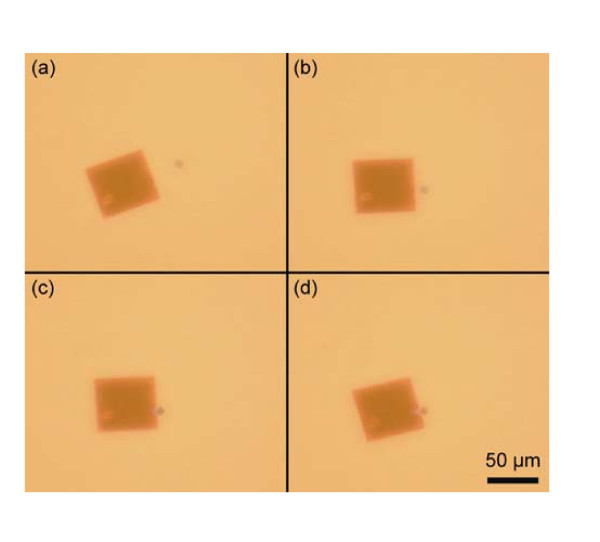
**Series of video frames showing attachment of a functionalized silica bead to a silicon nanomembrane**. The bead is false colored to improve image clarity. **(a) **The silica bead is trapped in three dimensions with a power of approximately 285 mW (shown immediately before the bead enters the trap). **(b) **The nanomembrane is moved toward the trapped bead using the microscope stage. **(c) **The bead is positioned immediately next to the nanomembrane and a bond forms, linking the two materials. **(d) **The nanomembrane rotates slightly in the plane into a preferred position with respect to the optical trap. The dimensions of the silicon nanomembrane are 50 × 50-μm, 220 nm thick.

Attachment of silica microsphere handles is shown in Figure 1. The nanomembranes were dispersed in a de-ionized water solution with trace amounts of isopropyl alcohol. 3 mL of this suspension was pipetted into an open sample well with a glass coverslip bottom. The membranes submerge to an equilibrium distance several hundred micrometers below the surface of the liquid. After a suitable membrane was identified, commercially available 10-μm diameter silica beads with an amine terminated (-NH_2_) surface functionalization (Sicastar^®^, Micromod Partikeltechnologie GmbH, Rostock-Warnemünde, Germany) were attached to the membrane using the optical trapping system. The functionalized microspheres, dispersed in de-ionized water, were added to the nanomembrane suspension. A single suspended bead was stably trapped at a power of approximately 300 mW and the submerged nanomembrane moved towards it using a Prior ProScan II (Rockland, MA, USA) motorized microscope stage. The bead was directed to near the desired attachment point and slowly brought to the edge of the nanomembrane using the microscope stage.

**Figure 2:**
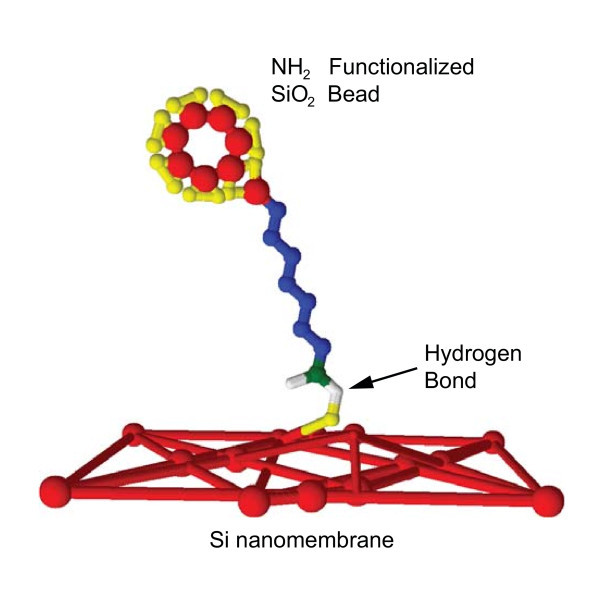
**Depiction of the bond that forms between the bead and the silicon nanomembrane**. A single bond is depicted for clarity. The bead surface is functionalized with -NH2 and the Si surface is oxidized.

As the microsphere is brought into the immediate vicinity of the desired location on the nanomembrane, a strong van der Waals attraction causes immediate attachment. The bead attaches to the side of the nanomembrane, as evidenced by both objects remaining in the same focal plane and the final location of the bead at the very edge. A thin layer of silicon dioxide readily forms on silicon in the presence of water or alcohols at room temperature [27]. The oxygen-terminated surface of the nanomembrane reacts at multiple sites with the hydrogen from the amine-functionalized bead. The resulting bond, shown in Figure 2 at a single attachment site for clarity, has a relatively weak energy of around 20 kJ/mol per bond [28] but is strong enough to allow the simultaneous manipulation of the microsphere and membrane without detachment. The multiple bonds are also strong enough to rotate the nanomembrane into an equilibrium position immediately upon attachment while the bead remains firmly fixed in the trap, also shown in the figure.

Once attached, the bead functions as a nanoscale trailer hitch, facilitating manipulation of the bead together with the nanomembrane. Using a single optical trap and the motorized stage, the nanomembrane can be translated laterally over millimeter distances with laser powers on the order of 200 to 700 mW as measured at the sample. Higher powers allow larger gradient forces and permit the trapped bead to be moved at greater velocities.

At powers higher than approximately 700 mW, we observe a break in the bond between the nanomembrane and microsphere, and the two structures become decoupled. Detachment is observed only if the laser power is greater than this minimum, and we reason that this results from laser heating in the immediate vicinity of the bead-silicon nanomembrane attachment site, weakening the coupling moiety, but we cannot at this stage determine where the break occurs. Most importantly, this decoupling process does not damage the nanomembrane and we can reattach the membrane to this or a new bead. The reversibility of our procedure is very encouraging for future work in nanomembrane assembly using optical tweezers.

In the above range of trapping powers, the nanomembrane can be transported using the optical tweezers/motorized-stage system at velocities reaching 200 μm/s. Above this limit, the microsphere and nanomembrane tandem fall out of the optical trap. We observe a similar maximum velocity when laterally translating identical beads without attachment to a nanomembrane, suggesting that the hydrodynamic drag resulting from the unique shape of the nanomembranes is minimal. An order-of-magnitude estimate of the fluid drag force on the planar nanomembrane is calculated from

(1) $$F_{\text{D}}=C_{\text{D}}\frac{V^{2}}{2}\rho A,$$

where *C*_D _is the drag coefficient for a rectangular sheet as a function of area and Reynolds number, *V *is the velocity, *ρ *is the density of the solution, and *A *is the surface area of the nanomembrane [29,30]. The resulting drag force at maximum velocity for the nanomembranes used in this work is approximately 0.5 pN, two orders of magnitude lower than the approximately 20 pN calculated for a 10-μm microsphere using Stokes's law, *F*_D _= 6πμRV [30]. We expect that this magnitude difference allows for lateral translation of nanomembranes having much larger surface areas, with minimal effect on motion at similar high velocities.

**Figure 3:**
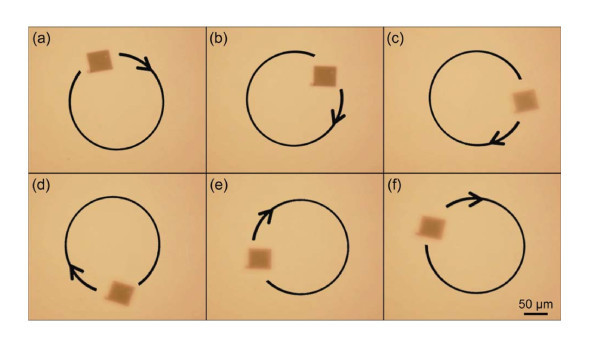
**Extracted video frames showing XY translation of an optically trapped silicon nanomembrane**. Frames **(a) **through **(f) **show the trapped membrane moved along a circular path in a single imaging plane. The path was defined using a custom built LabVIEW program to control the output of a spatial light modulator. The trapped membrane was translated along the path using discrete angular steps of 2.5°. When moving in a circular fashion, we see rotation of the nanomembrane around the trapped bead, stemming from the step-by-step directional change of fluid drag forces acting on the nanomembrane. Similar rotation is not observed when moving the nanomembrane in a straight line over long distances.

Finer-scale, more precise manipulation can be achieved using either diffracted traps from holographic optical tweezers or a steering mirror placed at a conjugate plane with respect to the objective lens. Figure 3 shows a trapped membrane being transported along a circular path that was predefined using the control software for the spatial light modulator. This motion occurs approximately 50 μm above the bottom of the sample well. Holographic optical tweezers offer exceptional control over individual trap positioning and velocity. Nanometer level manipulation at Angstrom-scale resolutions has been demonstrated for single optical traps [31], and this precision can be extended to multiple traps and multiple structures [32,33]. The benefits of this level of control are offset in part by the limited range of positioning that can be achieved within a single field of view of the ×20 microscope objective, approximately 350 × 450 μm. In comparison, the range afforded by our motorized microscope stage is several centimeters with 10-nm resolution and motion is limited only by the lateral dimensions of the sample well. Combining holographic optical tweezers with a motorized microscope stage offers unparalleled nanomembrane manipulation abilities over a large range of motion.

**Figure 4:**
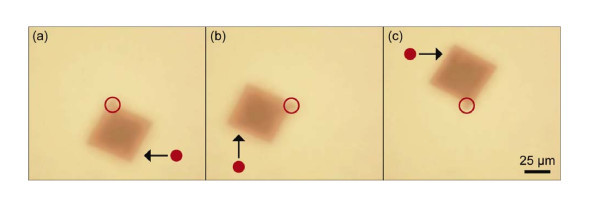
**Extracted video frames showing laser actuated rotation of an optically trapped silicon nanomembrane**. The silicon membrane is held in place using a single static trap directed at the attached functionalized bead. Frames **(a) **through **(c) **show membrane rotation about this coordinate using a second dynamic optical trap. The second trap does not directly interact with the nanomembrane but instead uses the optical scattering force to direct its motion. This indirect approach prevents damage to the strongly scattering silicon object.

We investigated other methods of membrane manipulation using holographic optical trapping abilities to demonstrate the versatility of this approach. We found that the in-plane orientation of a single nanomembrane can be controlled about a single point of rotation, by harnessing the deleterious scattering force that otherwise precludes direct manipulation of membranes without an attached bead. Figure 4 shows this rotation. The two-part structure is held in position using a stationary optical trap that secures the attached microsphere in place while a second dynamically generated holographic optical trap is used to direct and rotate the object in place. Once the second optical trap is removed, the membrane remains in its configured position barring heavy flow in the immediate vicinity. Thus the trapped microsphere functions as a kind of nanoscale hinge.

## Conclusions

In summary, we have used a functionalized-bead handle technique to translate and rotate high-lateral-size-to-thickness-aspect-ratio planar silicon nanomembranes in solution with holographic optical tweezers. The handle technique enables non-contact optical trapping of two-dimensional planar objects that could not otherwise be manipulated directly. Our approach permits individual nanomembrane positioning and transfer with unprecedented lateral control. The use of microspheres allows motion with well-documented nanometer-scale precision [14], while employing holographic optical trapping facilitates computer control of trajectories and enhanced positioning accuracy. We also demonstrated reversible attachment and detachment of handle beads without cutting or damaging the silicon material. We expect that this trapping method can be extended to manipulating silicon nanomembranes having larger lateral dimensions and differing thicknesses, in addition to the directed assembly of various other shapes and material compositions of planar objects with exceedingly small thickness [34-37]. The ability to tune the bond strength for membranes having different surface terminations may provide a future path for more selective, simultaneous manipulation of a variety of different planar materials. The successful use of holographic optical tweezers demonstrated here could be expanded to include massively parallel control over multiple nanomembranes, thus making heterostructure stacking and assembly a realizable goal. This simple proof of concept could eventually enable more advanced non-contact nanofabrication using nanomembranes as building blocks for two- and three-dimensional optical and electronic devices.

## Methods

### Fabrication of Si nanomembranes

The silicon nanomembranes, 220 nm thick, were fabricated from the template (outermost crystalline silicon) layer of commercially procured silicon-on-insulator (SOI) wafers (SOITEC S.A., Bernin, France) [38,39]. The wafers were cleaned with acetone, methyl alcohol, and isopropyl alcohol prior to patterning with electron beam lithography to define the square boundaries of each nanomembrane. Reactive ion etching was employed to etch the template silicon layer along these boundaries followed by a wet etch in 49% hydrofluoric acid for 4 h to dissolve the underlying SiO_2 _layer_. _The wet etch causes the patterned, thin silicon membranes to release and settle on the silicon handle wafer, the bottom layer of the SOI. The patterned membranes were then removed from the handle wafer by immersion in isopropyl alcohol and re-suspended in de-ionized water via a solvent exchange, where they remained stably dispersed for several weeks.

### Experimental system

**Figure 5:**
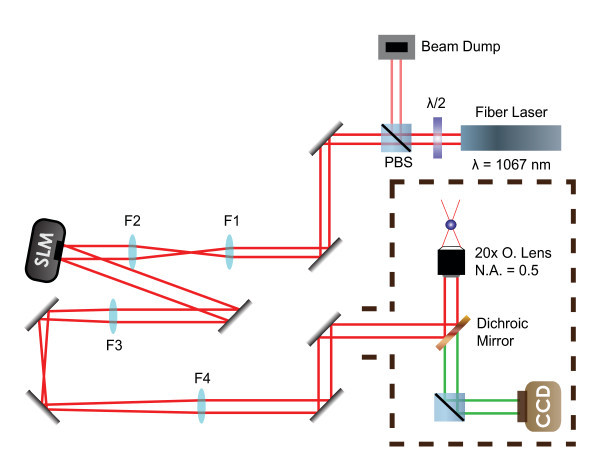
**Experimental setup**. The schematic diagram shows expansion of linearly polarized fiber laser light onto a spatial light modulator (SLM) using lenses F1 (*f *= 100 mm) and F2 (*f *= 200 mm). A second telescope (lenses F3 and F4, *f *= 500 mm) produces a plane conjugate to the surface of the SLM at the back aperture of the microscope objective (20x objective lens shown). CCD = charge coupled device, for recording images.

The holographic optical trapping system consists of a linearly polarized IPG Photonics YLR-10-1064-LP (Oxford, MA, USA) Ytterbium fiber laser operating at 1,067 nm with a maximum output power of 10 W. The laser is reflected off a Hamamatsu Photonics LCOS-SLM X10468 (Hamamatsu City, Japan) spatial light modulator (SLM), aligned through beam expanding optics, and finally directed into an Olympus IX71 inverted microscope (Olympus America, Inc., Center Valley, PA, USA), as shown in Figure 5. The beam diameter is expanded to slightly overfill the back aperture of the Olympus objective lens used for all experiments [26], ×20, numerical aperture (NA) = 0.5. Holographic optical traps were generated and dynamically controlled using a customized LabVIEW program adapted from a freeware SLM control code available from the University of Glasgow [40,41]. This interface gives us full three-dimensional spatial (translation and rotation) as well as temporal control over as many as 100 generated traps. Live power measurements were made continuously at the location of the beam dump, as shown in Figure 5, with more accurate measurements periodically made after the objective lens. All imaging was done using bright-field microscopy with a Lumenera Infinity 2 (Ottawa, Canada) digital microscope camera.

## Competing interests

The authors declare that they have no competing interests.

## Authors' contributions

SMO and JRSP performed the translation and manipulation experiments. RBJ and FSF fabricated the silicon membranes used in these studies. SMO, JRSP, and RJK participated in the design of the experiment; RJK and MGL supervised the work and prepared the manuscript. All authors read and approved the final manuscript.
